# STAT5 regulation of BCL10 parallels constitutive NFκB activation in lymphoid tumor cells

**DOI:** 10.1186/1476-4598-8-67

**Published:** 2009-08-26

**Authors:** Zsuzsanna S Nagy, Matthew J LeBaron, Jeremy A Ross, Abhisek Mitra, Hallgeir Rui, Robert A Kirken

**Affiliations:** 1 Department of Biological Sciences, University of Texas at El Paso, El Paso, TX 79968, USA; 2 Department of Cancer Biology, Thomas Jefferson University, Philadelphia, PA 19107, USA; 3 The Dow Chemical Company, Midland, MI 48674, USA

## Abstract

**Background:**

Signal Transducer and Activator of Transcription 5 A and B (STAT5) are key survival factors in cells of the lymphoid lineage. Identification of novel, tissue-specific STAT5 regulated genes would advance the ability to combat diseases due to aberrant STAT5 signaling. In the present work a library of human STAT5 bound genomic elements was created and validated.

**Results:**

Of several STAT5 responsive genomic regulatory elements identified, one was located within the first intron of the human *BCL10 *gene. Chromatin immuno-precipitation reactions confirmed constitutive *in vivo *STAT5 binding to this intronic fragment in various human lymphoid tumor cell lines. Interestingly, non-phosphorylated STAT5 was found in the nuclei of Kit225 and YT cells in the absence of cytokine stimulation that paralleled constitutive NFκB activation. Inhibition of the hyperactive JAK3/STAT5 pathway in MT-2 cells via the Mannich-base, NC1153, diminished the constitutive *in vivo *occupancy of BCL10-SBR by STAT5, reduced NFκB activity and BCL10 protein expression in a dose dependent manner. Moreover, depletion of STAT5 via selective antisense oligonucleotide treatment similarly resulted in decreased BCL10 mRNA and protein expression, cellular viability and impaired NFκB activity independent of IL-2.

**Conclusion:**

These results suggest that the NFκB regulator BCL10 is an IL-2-independent STAT5 target gene. These findings proffer a model in which un-activated STAT5 can regulate pathways critical for lymphoid cell survival and inhibitors that disrupt STAT5 function independent of tyrosine phosphorylation may be therapeutically effective in treating certain leukemias/lymphomas.

## Background

The family of mammalian Signal Transducer and Activator of Transcription (STAT) molecules is composed of 7 members (STAT1–4, 5A, 5B and 6) which mediates a variety of cellular processes including proliferation, differentiation and survival (reviewed in [1]). Current dogma suggests that STATs are latent factors residing in the cytosol that only become activated following ligand binding to receptors that initially results in the recruitment and activation of Janus tyrosine kinases (JAKs). JAKs then phosphorylate tyrosine residues on the receptor that serve as docking sites for SH2 domain-containing STATs and other signaling molecules. STATs subsequently become tyrosine phosphorylated by JAKs or other tyrosine kinases, disengage from the receptor, form dimers via phosphotyrosine-SH2 domain interactions, and translocate to the nucleus to initiate gene transcription [2,3].

Mammalian STATs can be classified based in parts on their function in promoting various cellular processes. For example, STATs 2, 4 and 6 are critical for the immune system to promote viral defense and Th1 versus Th2 differentiation, respectively. Conversely, STATs 1, 3, 5A and 5B are generally utilized by cytokines and growth factors that promote cellular growth, proliferation or death (reviewed in [1]). The members of this second group are associated with cancer formation, including STAT1 [4]. Intriguingly, STAT3 and STAT5 promote cell survival through shared target genes, including *Bcl-x *and *Pim-1 *[5-7]. Mice devoid of *Stat5a *and *Stat5b *genes have further established these proteins as important regulators of T-cell function [8,9]. Interestingly, IL-2 induced T cell proliferation was markedly affected only when both *Stat5a *and *Stat5b *genes were inactivated suggesting that they play redundant roles [9]. In addition to lymphocytes, STAT5A and STAT5B act as major survival factors for several cell types including mammary epithelium [10,11] and human prostate cancers [12]. Cancer cells from certain lymphomas and leukemias also display hyper tyrosine-phosphorylated STAT5 as a result of chromosomal translocations, deregulated tyrosine kinases or viral transformation as reviewed in [1].

Chromatin immuno-precipitation has been a widely utilized method to study direct transcription factor-DNA interactions [13] and for identifying transcription factor binding sites in unknown target genes by cloning captured DNA material [14] generated from a genome-wide library that ultimately can be sequenced and located. Alternatively, captured DNA material can be hybridized to microarrays representing (i) CpG rich regions of a genome that are contained in a significant portion of promoter elements [15] or (ii) non-coding regions within whole chromosomes [16]. Both of these aforementioned methods have shed new light onto the biological function, location and kinetics of transcription factor/DNA binding dependent gene expression.

The present study was designed to identify genome-wide immune specific STAT5 regulated genes. This approach has shown promise in identifying STAT5 target genes in mouse pro-B cells [17] and human prolactin treated T47-D breast cancer cells [18]. A library of STAT5-bound genomic fragments was created by cloning and sequencing chromatin immuno-precipitated DNA fragments from the human lymphoma cell line, YT. One of these sequences was identified within an intronic element of the *BCL10 *gene. We showed that STAT5 constitutively occupied this region *in vivo *in multiple human lymphoid cell lines. Intriguingly, non-phosphorylated STAT5 was present in the nuclei of lymphoid cells that paralleled constitutively active NFκB. Disrupting JAK3 activity diminished the *in vivo *binding of STAT5 to BCL10-SBR in MT-2 cells, reduced NFκB activity and BCL10 protein expression. Furthermore, specific STAT5 depletion correlated with decreased NFκB DNA-binding, cell viability and BCL10 protein expression in both the presence and absence of IL-2. Taken together, these findings indicate a novel cross-talk mechanism between the STAT5 and NFκB pathways.

## Results and Discussion

### Generation of a library encoding STAT5 Binding Regions

**Figure 1:**
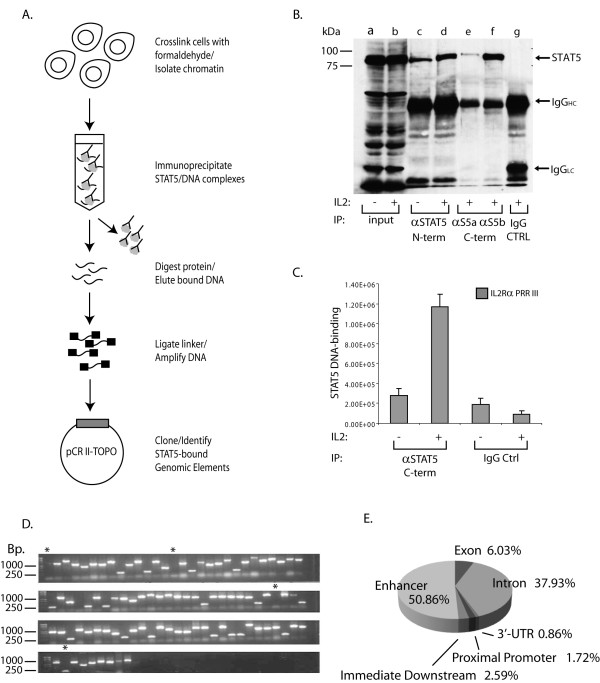
**(A) Generation of a library encoding STAT5-responsive genomic elements by ChIP-cloning**. IL-2 stimulated, formaldehyde cross-linked YT cells were lysed, sonicated and immuno-precipitated with antibodies to STAT5A or STAT5B. Eluted DNA was ligated to a unidirectional linker (black blocks), amplified and then cloned into pCR II-TOPO vector. Clones containing inserts were identified by sequencing. (B) **Successful immuno-precipitation of STAT5 from formaldehyde fixed YT cell lysates**. YT cells were stimulated with medium (-) or IL-2 (+) then fixed with formaldehyde. Fixed lysates were immuno-precipitated with antibodies to STAT5 as indicated or normal rabbit serum (IgG CTRL) then Western blotted for STAT5. Molecular weight markers are indicated to the left side of the panel. Input material corresponds to 1% of cell lysate used in the immuno-precipitations. (C) **Validation of STAT5 ChIP in YT cells**. ChIP assay with C-terminal antibodies to STAT5A and B in combination (αSTAT5 C-term) or IgG control was carried out as described above. The eluted DNA was then used as template in qPCR reactions with primers designed to PRR III. (D) **STAT5 bound genomic library captured by ChIP-cloning**. Inserts were amplified via PCR using M13 primers prior to sequencing and visualized by agarose gel electrophoresis (1%). Stars (*) indicate clones without an insert. (E) **Nearby gene mapping of the ChIP-clone identified genomic sequences**. One hundred and nineteen clones were sequenced, 3 fragments were duplicates and 9 were greater than 300 kb away from any coding region. The remaining sequences that fell within 300 kb from coding regions were analyzed with Cis-Regulatory Element Annotation System (CEAS). The pie chart represents "%" distribution.

Since STAT5 is critical for maintaining lymphoid cell survival [1], we sought to identify putative target genes that could be responsible for this phenotype. In the present work a lymphoma-specific library of IL-2-induced STAT5 bound genomic elements was generated by cloning chromatin immuno-precipitated genomic sites directly occupied by STAT5 as described in Figure 1A and in the Methods.

### Validation of STAT5 chromatin immuno-precipitation in YT cells

In order to confirm that STAT5 was successfully immuno-precipitated from formaldehyde-treated chromatin, YT cells were stimulated with medium (-) or IL-2 (+) for 30 min at 37°C then fixed with formaldehyde. Next, STAT5 was immuno-precipitated with antibodies raised against the N-terminus (recognizes both STAT5A and STAT5B, lanes c-d) or the C-terminus of either STAT5A (lane e) or STAT5B (lane f), or normal rabbit serum as control IgG (lane g), separated by SDS-PAGE, and subsequently Western blotted with monoclonal anti-STAT5 antibody. Whole cell lysate (1% of IP) was also loaded to demonstrate equal input material for immuno-precipitation (lanes a-b). As shown in Figure 1B, all three antibodies were competent to bind STAT5 (indicated by arrow to the right) from fixed cells as compared to the control (lane g). To confirm successful capture of genomic elements known to be occupied by STAT5, qPCR reactions were performed with primers designed to the region harboring a known STAT5 binding site within the human *IL2RA *enhancer (Positive Regulatory Region PRR III; Figure 1C) [19]. Data presented in Figure 1C indicated that STAT5 antibody successfully enriched PRR III as compared to control IgG (nrs).

Next, a library containing STAT5 bound genomic fragments was created by amplification and cloning ChIP-ed DNA material as described in the Methods. The colonies were tested for the presence of inserts by direct PCR amplification using vector specific M13 primers (representative colonies shown in Figure 1D) prior to sequencing. One hundred and nineteen clones were sequenced and the genomic locations analyzed with nearby gene mapping (CEAS) as described in the Methods. Genomic allocation of the clones is depicted in Figure 1E demonstrating the majority of the identified sequences were found in intronic (38%) and enhancer (51%) regions. These data are in agreement with earlier findings that binding sites of transcription factors are not restricted to promoter regions, rather, a significant portion of these sites are present in introns [16].

### Validation of putative STAT5 binding genomic regions by EMSA-cold competition assays

**Figure 2:**
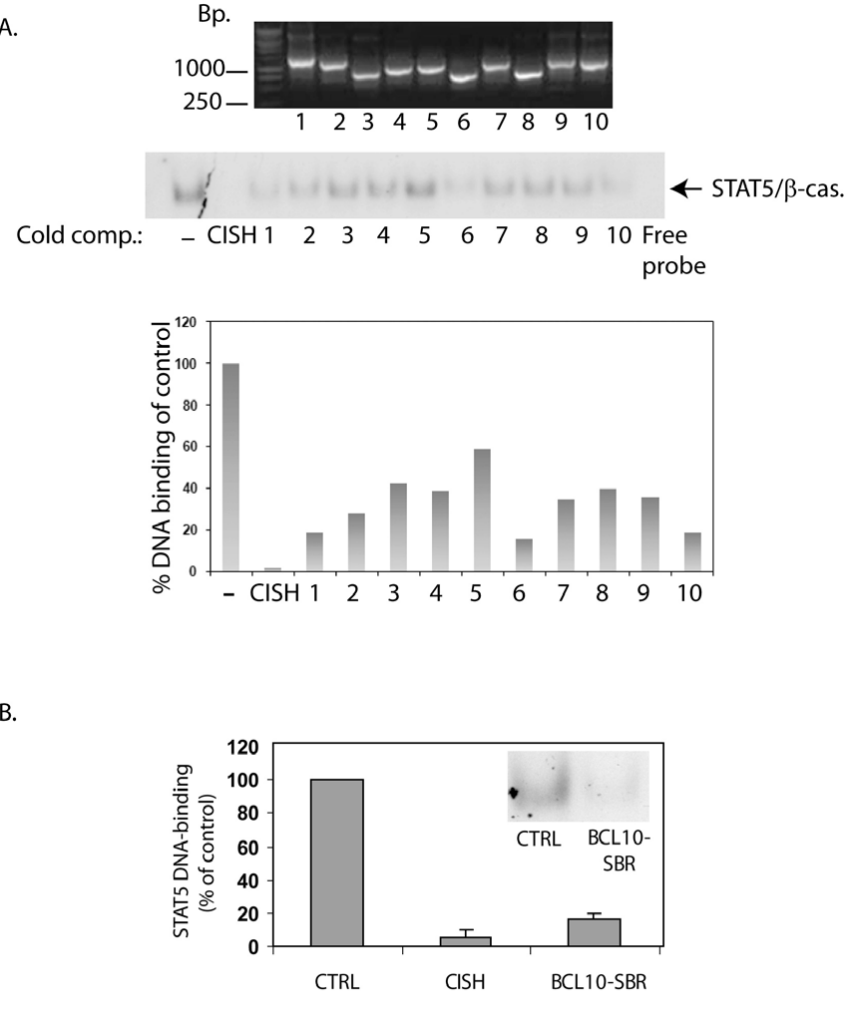
**(A) Validation of the STAT5 genomic library by cold competition EMSA analysis**. Ten randomly selected STAT5 bound genomic fragments were amplified with M13 primers (upper panel) and used as cold competitors at 30–50-fold molar excess in EMSA assays using IL-2 stimulated YT nuclear extracts and [^32^P]-labeled STAT5-probe (lower panel). DNA-binding was expressed as % of control (a reaction without cold competitor (-)) as shown in the graph. The PCR amplicon surrounding the STAT5 binding site in the enhancer of the human *CISH *gene was used as a positive control. (B) **Validation of STAT5 binding to BCL10-SBR**. PCR amplified BCL10-SBR was used as a cold competitor in EMSA assays employing IL-2 stimulated YT nuclear extracts and [^32^P]-labeled STAT5-probe (a representative of a BCL10-SBR cold competition EMSA analysis is shown). Band intensities were determined by densitometric analysis. The results presented are an average of two independent experiments for BCL10-SBR and three for CISH.

To confirm that clones encoding the sequenced genomic elements (Figure 1D) can be bound by STAT5, inserts from randomly selected colonies were amplified (from 52 clones, 10 representatives shown in Figure 2A, upper panel) and used in 30–50-fold molar excess as cold competitors in EMSA assays employing [^32^P]-labeled probe corresponding to the STAT5 binding site in the *β-casein *gene promoter and nuclear extracts from IL-2 stimulated YT cells (Figure 2B, lower panel) [14]. The results were quantitated by comparing the band intensities of the cold competition EMSA reactions to the control reaction (as shown in Figure 2A graph). Of 52 validated clones, 24 fragments caused greater than 50% decrease in STAT5 DNA-binding intensity to the radioactively labeled probe. Table 1 summarizes the genomic location of the 20 validated clones located within 300 kb of coding sequences as performed by CEAS (four genomic segments were further than 300 kb from any coding regions).

**Table 1:** Nearby gene mapping of validated ChIP-cloned sequences.

Chr	Start	End	% DNA-b.	Strand	Dir.	Gene Name	Loc.
1	212539607	212540208	40.79	+	U	SMYD2	I
				-	U	PTPN14	E

1	184551050	184551638	46.99	+	U	PRG4	IDS
				+	D	C1orf27	E
				-	U	TPR	I

1	148762360	148762960	17.71	+	U	ECM1	E
				+	D	ADAMTSL4	E
				-	U	C1orf138	E
				-	D	APH1A	E

1	85509312	85509912	18.52	+	U	WDR63	E
				-	U	BCL10	I
				-	D	C1orf52	E

1	172086277	172086887	3.81	+	U	DARS2	I
				+	D	ZBTB37	E
				-	U	SERPINC1	E
				-	D	CENPL	E

1	210194877	210195427	35.81	+	D	DTL	E
				-	U	INTS7	I
				-	D	LPGAT1	E

2	38751513	38752103	47.52	+	U	GALM	I
				+	D	GEMIN6	E
				-	U	SFRS7	E
				-	D	HNRPLL	E

3	140069314	140069963	5.83	+	U	FAIM	E
				-	U	FOXL2	E
				-	D	PIK3CB	E

4	77775984	77776232	18.02	+	U	SHRM	I
				-	U	LOC345079	E
				-	D		E

12	12538539	12539089	13.72	+	U	LOH12CR1	E
				+	D	CREBL2	E
				-	U	DUSP16	I
				-	D	MANSC1	E

12	22082357	22083007	41.9	+	D	CMAS	E
				-	U	ST8SIA1	E
				-	D	ABCC9	E

12	88442012	88442661	26.03	-	U	WDR51B	I
				-	D	DUSP6	E

13	102554697	102555277	9.23	+	U	ERCC5	E
				-	D	SLC10A2	E

13	73026115	73026665	34.44	-	U	KLF12	E

14	68177078	68177617	35.78	+	U	RAD51L1	E
				-	U	ZFP36L1	E

15	24494535	24495711	32.64	+	D	GABRA5	E
				-	U	GABRB3	I

16	73675183	73675822	40.32	+	U	ZNRF1	I
				+	D	ZFP1	E
				-	U	LDHD	E
				-	D	WDR59	E

16	16389501	16389952	43.06	+	U	NOMO3	E
				-	D	ABCC6	E

19	19238022	19238623	13.61	+	U	CSPG3	E
				+	D	KIAA0892	E
				-	U	TM6SF2	E
				-	D	HAPLN4	E

X	149544804	149545374	27.66	+	U	MTM1	I
				+	D	MTMR1	E
				-	U	CD99L2	E

Randomly selected sequences (52) were validated by cold competition EMSAs as described in the Methods. Clones (24) with greater than 50% decrease in STAT5 DNA-binding to the [^32^P]-labeled probe were analyzed using CEAS. Fragments within 300 kb from coding sequences (20) are summarized above. (IDS: Immediate Downstream; U: Upstream; D: Downstream; I: Intron; E: Enhancer; chr: chromosome; dir: direction; % DNA-b.: % DNA-binding)

### STAT5 binds an intronic element within the human *BCL10 *gene *in vitro*

One putative STAT5 responsive region was identified within the first intron of the *BCL10 *gene, a known regulator of NFκB activity and an essential positive regulator of T and B cell development and activation [20]. The *BCL10 *gene is located on chromosome 1 and is composed of four exons and three introns. The STAT5 binding region was confined to the second intron, proximal to the 5' end of the third exon which we designated as the BCL10-STAT5 Binding Region (BCL10-SBR). To confirm this finding, PCR amplified BCL10-SBR was used as a cold competitor in EMSA assays as described above. Data from two independent experiments (Figure 2B) showed that BCL10-SBR reduced STAT5 binding to the radioactively labeled probe greater than 80% suggesting that this element was bound by STAT5 *in vitro*. The genomic region surrounding the STAT5 binding site in the human CISH promoter was also amplified and used as a positive control. BCL10 is an adapter molecule implicated in antigen receptor-mediated NFκB signaling by linking to the IκB kinase complex. The relevance of BCL10 mediated NFκB signaling for lymphoid cells has been described in *Bcl10 *deficient mice as T and B cells derived from these animals are nonfunctional and exhibit impaired B/T cell receptor signaling, as a consequence of impaired NFκB signaling [20,21]. These results suggest an intriguing cross-talk between the STAT5 and NFκB pathways, which are both implicated in malignant transformation. [1,22]

### STAT5 constitutively occupies BCL10-SBR *in vivo*

**Figure 3:**
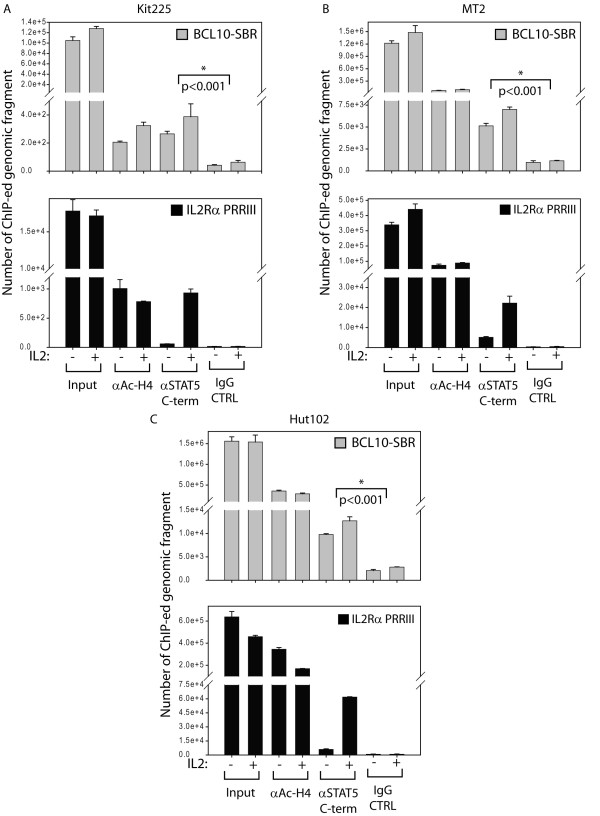
**STAT5 constitutively occupies BCL10-SBR *in vivo *in Kit225, MT2 and Hut102 cells**. Kit225 cells (A) were made quiescent in medium without IL-2 for 24 h, MT2 (B) and Hut102 (C) cells were grown until exhaustion. Cells were then stimulated with medium (-) or IL-2 (+) for 30 min at 37°C, then fixed with 1% formaldehyde for 10 min at room temperature and then chromatin immuno-precipitated with antibodies to Acetyl-Histone 4, C-terminal STAT5 or control IgG. The eluted DNA was amplified with primers corresponding to PRR III (black bars) or BCL10-SBR (grey bars). Representative data for MT2, Hut102 (n = 2), and Kit225 cells (n = 3) are shown. Input material represents 5% of immuno-precipitated chromatin.

Cold competition EMSA assays indicated that BCL10-SBR can bind STAT5 *in vitro*. Next, we sought to test whether STAT5 can also bind this genomic element *in vivo*. For this analysis, ChIP assays were performed with antibodies to STAT5 (C-terminal), acetylated-Histone 4 antibody (α H4-Ac, to confirm active transcription at these sites [23]) or control IgG (normal rabbit serum) in un-stimulated (-) or IL-2-stimulated (+) Kit225 (Figure 3A), MT2 (Figure 3B) and Hut102 (Figure 3C) cells. Bound DNA was eluted and amplified with primers specific to PRR III or BCL10-SBR via qPCR. Indeed, IL-2-inducible enrichment of PRR III occurred with the STAT5 C-terminal antibody (lower panels). Intriguingly, *in vivo *binding of STAT5 to BCL10-SBR was demonstrated in an IL-2-independent manner in all three cell lines examined (upper panels). These results demonstrate that STAT5 constitutively occupies BCL10-SBR *in vivo*. However, IL-2-induced enrichment of the STAT5-responsive PRR III showed that STAT5 was able to bind DNA in a tyrosine-phosphorylation dependent manner as well in these cell lines. Earlier studies with STAT1 indicated that non-phosphorylated STAT1 had unique genomic binding sites [24]. Based on these results it may be logical to assume that non-phosphorylated and phosphorylated (cytokine independent and dependent, respectively) STAT5 might have unique target sites, different binding characteristics, and perhaps binding partners.

### STAT5 is localized to the nucleus of YT and Kit225 cells in the absence of cytokine stimulation

**Figure 4:**
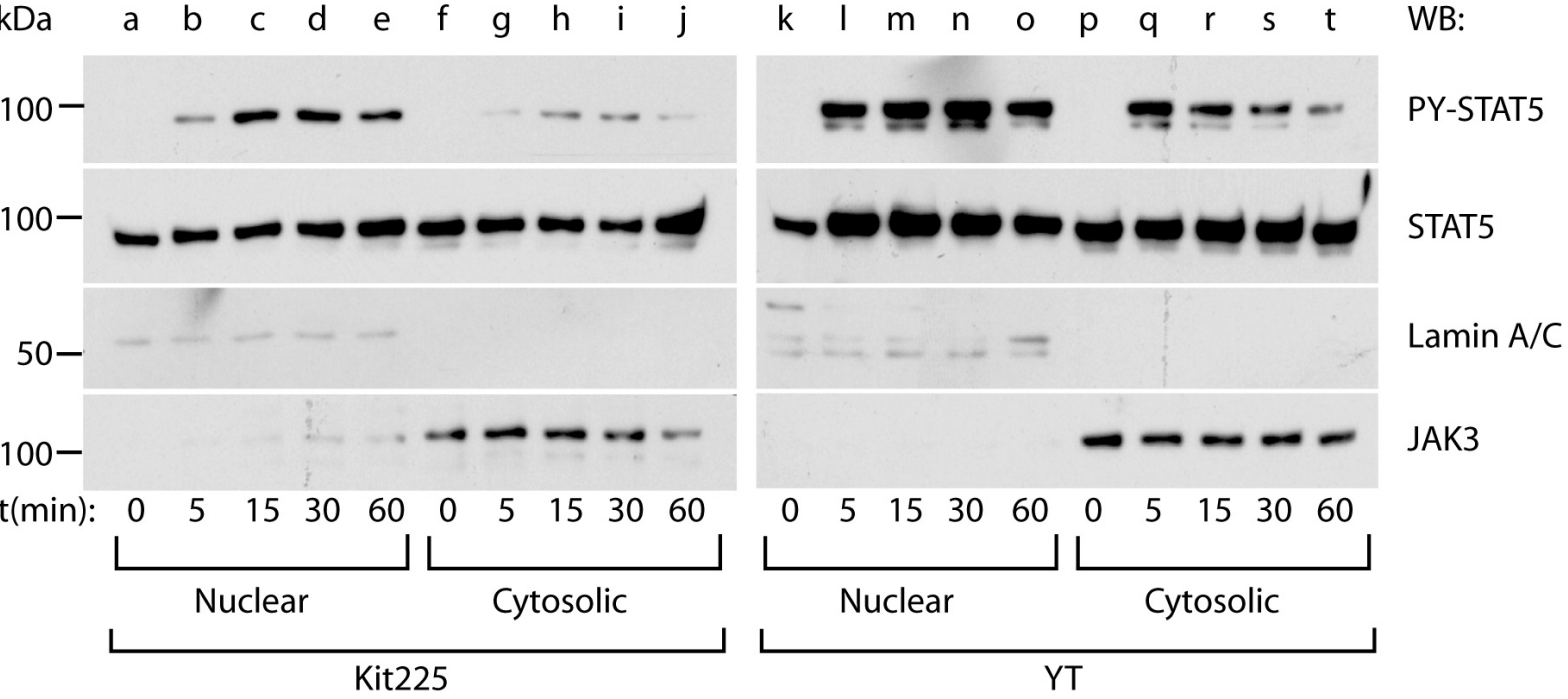
**Nuclear localized STAT5 is present in YT and Kit225 cells in the absence of IL-2 stimulation**. Equal amounts (10 μg) of nuclear and cytosolic proteins (Kit225: lanes a-j, YT: lanes k-t) were resolved on a 10% SDS-PAGE and Western blots performed with PY-STAT5, STAT5, Lamin A/C (nuclear marker) or Jak3 (cytosolic marker) antibodies (indicated to the right). Representative data from three independent experiments are presented.

**Figure 5:**
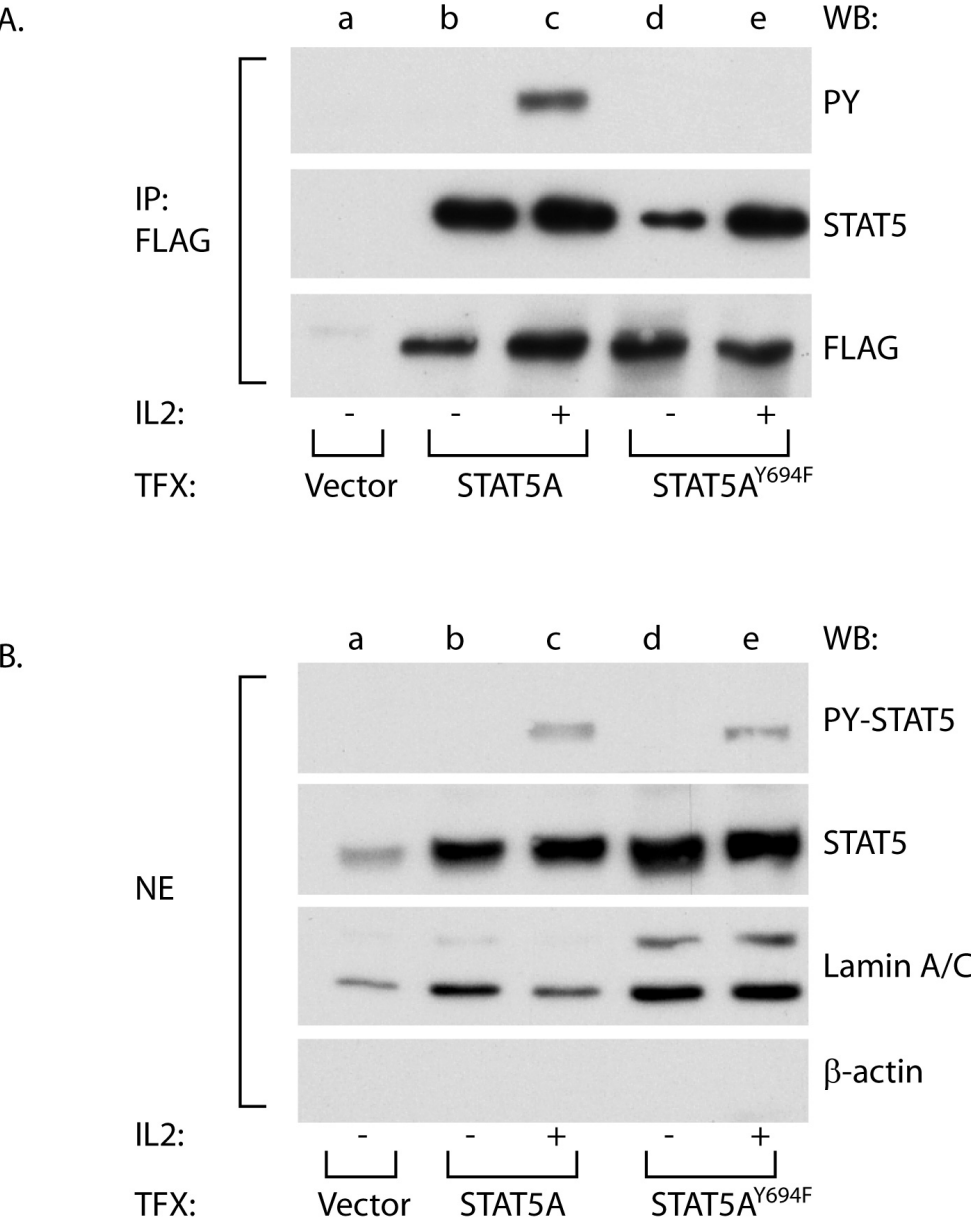
**Y694F-mSTAT5A can localize to the nuclei of YT cells**. (A) YT cells over-expressing vector alone (Vector), wt or Y694F mSTAT5A were stimulated with medium (-) or IL-2 (+) for 30 min at 37°C. Nuclear extracts were prepared and immuno-precipitated with anti-FLAG antibodies, resolved on 7.5% SDS-PAGE then Western blotted with PY antibodies followed by re-blotting with antibodies to STAT5 and FLAG as indicated to the right. (B) Nuclear extracts isolated as described above were resolved on a 7.5% SDS-PAGE, Western blotted with PY-STAT5 antibody then re-blotted with antibodies to STAT5, Lamin A/C and β-actin as indicated to the right.

Current models hold that tyrosine phosphorylated STAT dimers are required for gene regulation. However, new evidence suggests that STAT proteins traffic to the nucleus and regulate gene expression independent of tyrosine phosphorylation [24-26]. Indeed, data presented in Figure 3 indicated that STAT5 can bind to BCL10-SBR in a constitutive manner in three cell types tested in the absence of IL-2. To confirm this hypothesis, nuclear and cytosolic proteins were isolated from Kit225 (lanes a-j) and YT cells (lanes k-t) stimulated with IL-2 for the times indicated, equal amounts (10 μg) of proteins were separated on 10% SDS-PAGE and Western blotted with PY-STAT5 antibody (specifically recognizes phosphorylated Y694 and 699 for STAT5A and B, respectively) followed by re-probing the membrane for total STAT5. Antibodies to Lamin A/C (nuclear marker) and JAK3 (cytosolic marker) were employed to confirm the purity of the extraction. As shown in Figure 4, non-phosphorylated STAT5 was present in the cell nuclei in the absence of IL-2 stimulation. However, IL-2 was able to induce accumulation of tyrosine-phosphorylated STAT5 in the nuclear fraction. These data suggest that the presence of STAT5 in the nuclei is not dependent on its tyrosine phosphorylation status. To further demonstrate that non-tyrosine-phosphorylated STAT5 can localize to the nuclear compartment in lymphoid cells, wild type (wt) or Y694F mutant of mSTAT5A (unable to undergo tyrosine phosphorylation [27]) were N-terminally FLAG-tagged and over-expressed in YT cells as described in the Methods. Next, nuclear extracts were prepared from cells over-expressing vector alone (Figure 5A, lane a), wt (lanes b-c) or Y694F mSTAT5A (lanes d-e) stimulated with medium (-) or IL-2 (+) for 30 min at 37°C as indicated. Nuclear extracts were immuno-precipitated with anti-FLAG antibodies then Western blotted with antibodies to PY (Fig. 5A, upper lane), STAT5 (middle lane) or FLAG (lower lane). While wt mSTAT5A was tyrosine-phosphorylated upon IL-2 stimulation, the Y694F mutant was not. However, both wt and Y694F mSTAT5A were constitutively present in the cell nuclei suggesting that STAT5 nuclear localization can occur in the absence of tyrosine phosphorylation. To confirm that YT cells over-expressing Y694F mSTAT5A retained the ability to respond to IL-2, as well as to demonstrate that STAT5 nuclear presence was not due to contamination with cytosolic proteins, whole nuclear extracts isolated above were Western blotted with PY-STAT5 then re-blotted with antibodies to STAT5, Lamin A/C (a nuclear marker) followed by β-actin (cytosolic marker) as shown in Figure 5B. Similar results were obtained with Y699F mSTAT5B (data not shown).

Traditionally, STAT transcription factors were thought to reside in the cytoplasm in the absence of cytokine stimulation, and only enter the nucleus to bind DNA and initiate gene expression following cytokine engagement [2,28]. However, interesting new evidence suggests that nuclear-localized non-tyrosine-phosphorylated STATs can regulate gene expression. Indeed, interferon-mediated gene expression changes in a STAT1-deficient cell line transfected with a Y699A mutant of STAT1 unable to become tyrosine-phosphorylated proved it can initiate constitutive gene expression [24]. Other recent publications have reported that STAT3 can also induce gene transcription in the absence of tyrosine phosphorylation [26]. Moreover, non-phosphorylated, nuclear localized STAT6 in a non-small cell lung cancer model was shown to drive cyclooxygenase-2 expression independent of its tyrosine phosphorylation status [25]. Our results provide the first evidence that non-tyrosine-phosphorylated, nuclear-localized STAT5 may also play a similar and critical role in gene regulation in lymphoid cells in the absence of stimulation/activation.

### NFκB is constitutively active in YT, Kit225 cells and activated human PBMCs

**Figure 6:**
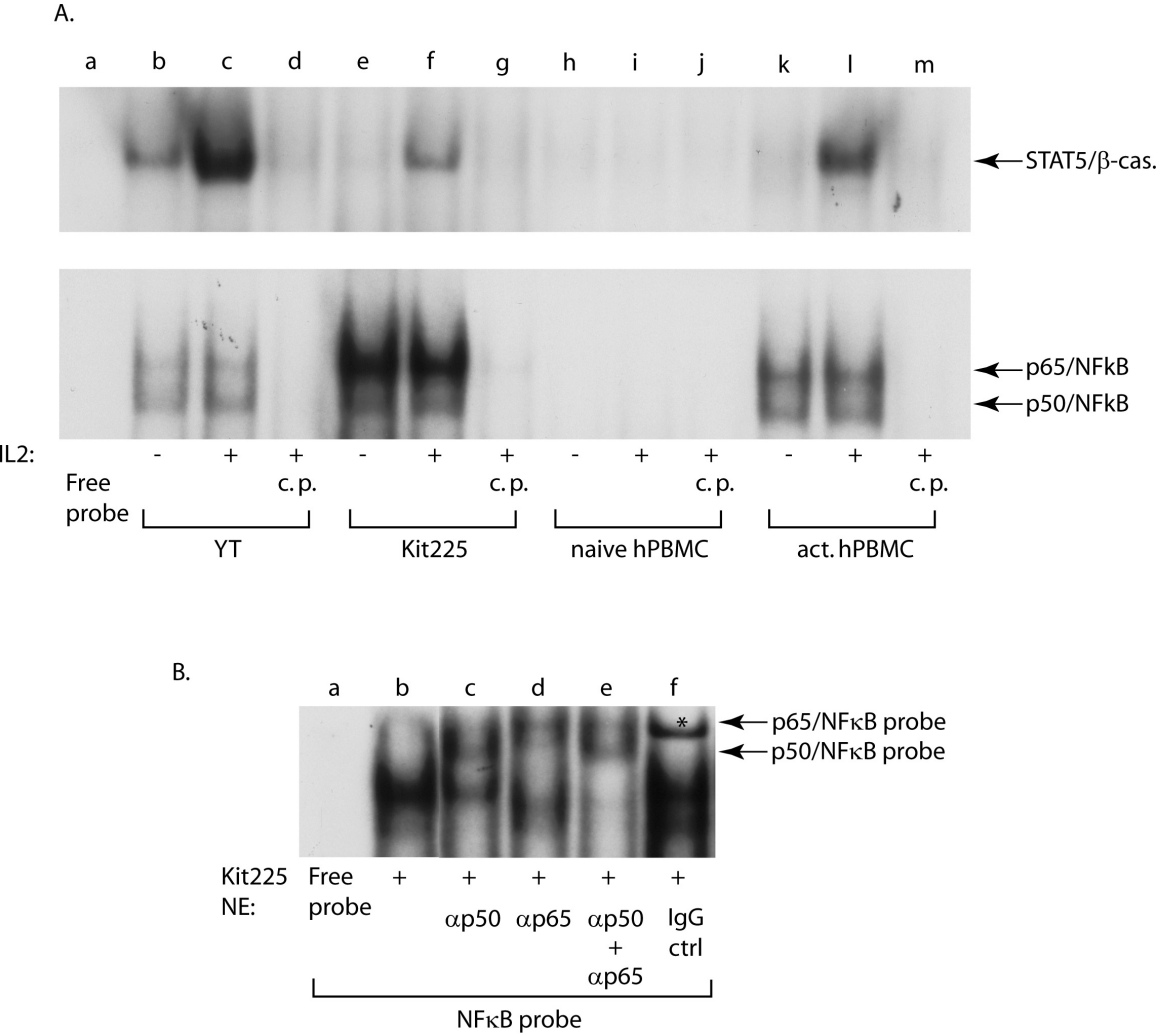
**NFκB is constitutively active in multiple lymphoid cells **(A) YT (lanes b-d), quiescent Kit225 (lanes e-g), naïve (lanes h-j) and quiescent activated human PBMCs (lanes k-m) were stimulated with IL-2 for 30 min before nuclear extracts were prepared. Equivalent amounts (5 μg) were used in EMSA reactions employing a STAT5 (upper panel) or NFκB radio-labeled probe (lower panel). A reaction without nuclear extract (free probe, lane a) or with unlabeled probes (lanes d, g, j, m) were used to confirm the specificity of the bands. (B) **Constitutive NFκB is composed of p50 and p65 subunits in lymphoid cells**. Antibodies to p50 (lane c) and p65 (lane d) subunits of NFκB were used either alone or in combination (lane e) for supershift assays in Kit225 nuclear extracts. Control IgG (normal goat serum, lane f) and a reaction without nuclear extract (free probe, lane a) were used to confirm the specificity of the bands. Star (*) indicates a non-specific band.

Since BCL10 is a positive regulator of NFκB [20], next we sought to test the activation status of NFκB in lymphoid cells. EMSA analysis was performed with either a [^32^P]-labeled NFκB (lower panel) or STAT5 (upper panel) probe and 5 μg nuclear extracts from YT (Figure 6A, lanes b-d), Kit225 (lanes e-g) cells or naïve (lanes h-j) and activated (lanes k-m) human PBMCs stimulated with medium (-) or IL-2 (+) for 30 min. Figure 6A demonstrated that while IL-2 was able to induce DNA-binding of STAT5 in YT (lane c), Kit225 (lane f) and PBMCs (lane l), NFκB DNA-binding was constitutive in these cells. Naïve PBMCs, which did not respond to IL-2, did not display binding to either probe, thus verifying that constitutive NFκB binding was not an artifact resulting from nuclear extraction. To confirm the specificity of the observed bands, a reaction without nuclear extract (Free probe) and cold competition assays with the corresponding unlabeled probes (c.p.) were also performed (lanes d, g, j and m). To further verify the specificity of the NFκB bands, antibodies to p50 (Figure 6B, lane c), p65 (lane d) or both (lane e) were used in supershift analyses. Indeed, both p50 and p65 antibodies resulted in partial supershifts of the NFκB band, while using these antibodies in combination resulted in a complete supershift. On the contrary, normal goat serum (IgG ctrl, lane f) did not result in a supershift of the NFκB bands.

### Blockade of the JAK3/STAT5 pathway diminishes *in vivo *STAT5 binding to BCL10-SBR, impairs NFκB function and reduces BCL10 expression

**Figure 7:**
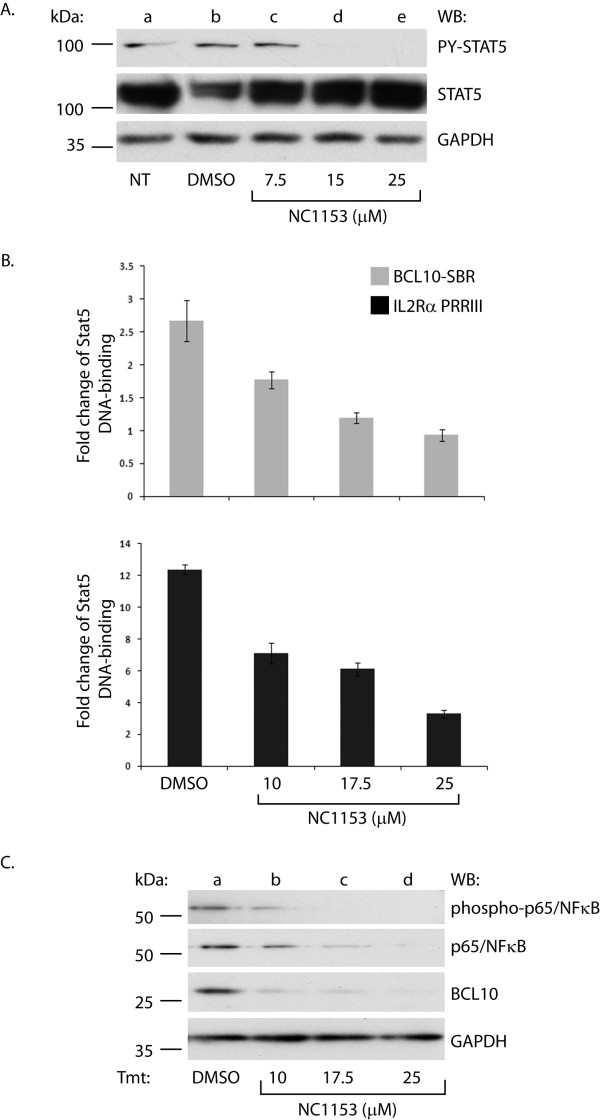
**JAK3/STAT5 blockade inhibits constitutive STAT5 tyrosine phosphorylation and BCL10/NFκB activity in MT-2 cells**. (A) MT-2 cells were treated with medium, DMSO (0.1%, vehicle control) ascending amounts of NC1153 as indicated for 24 h. Total cell lysates were Western blotted with antibodies as indicated to the right. Molecular weight markers are indicated to the left. (B) **JAK3 blockade diminishes BCL10-SBR occupancy by STAT5 *in vivo***. MT-2 cells were treated with NC1153 as indicated for 10 h. ChIP assays were performed with anti-STAT5 antibody and IgG control from each treatment. STAT5 DNA binding is expressed as fold change over background (IgG control). (C) **NC1153 reduces NFκB activity and BCL10 protein expression**. MT-2 cells were treated as described above for 48 h. Total cell lysates were Western blotted with antibodies indicated to the right. Molecular weight markers are shown to the left.

In order to confirm that the *in vivo *binding of STAT5 to BCL10-SBR is responsive to the inhibition of the JAK3/STAT5 pathway, we employed the selective JAK3 inhibitor NC1153 [29]. Although the precise regulation of STAT5 by JAK3 is not yet fully understood, it has been shown that phosphorylated STAT1 and STAT3 can increase the expression of non-phosphorylated STAT1 and STAT3, respectively [30]. Therefore, it was hypothesized that non-phosphorylated STAT5 function could partially be affected by the inhibition of phosphorylated STAT5. First, the activation status of the JAK3/STAT5 pathway was tested in MT-2 cells treated with ascending amounts of NC1153 for 24 h as indicated (Figure 7A, lanes c-e) by Western blotting. Constitutive tyrosine phosphorylation of STAT5 was diminished by NC1153 in a dose dependent manner as compared to non-treated (NT, lane a) or vehicle treated (DMSO, lane b) samples. Equal loading was confirmed by re-probing the membrane with antibodies to STAT5 and GAPDH. Moreover, tyrosine phosphorylation of JAK3 was similarly decreased upon NC1153 treatment (data not shown). Next, *in vivo *binding of STAT5 to PRR III and BCL10-SBR were assessed by ChIP assays and qPCR. As presented in Figure 7B, the occupancy of these regions by STAT5 was reduced in a dose dependent manner upon NC1153 (expressed as fold change of STAT5 binding over background (IgG control)). Lastly, the functional effect of JAK3 blockade on the expression of BCL10 protein and the activation status of NFκB was assessed. Since BCL10 is a known regulator of NFκB signaling in lymphoid cells [20] that is a critical pathway for mediating survival of activated B- and T-cells, it was reasonable to assume that STAT5 depletion mediated decrease of BCL10 expression might lead to diminished constitutive NFκB activation. For this assay, MT-2 cells were treated with DMSO (Figure 7C, lane a) or ascending concentrations of NC1153 for 48 h as indicated (lanes b-d), then harvested and Western blotted with antibodies to phospho-p65/NFκB, p65/NFκB and BCL10. Indeed, data presented in Figure 7C demonstrated that phosphorylation of p65 NFκB on Ser536, an indicator of its enhanced transcriptional activity [31], was decreased in parallel to BCL10 protein expression upon NC1153 treatment. Equal loading was confirmed by re-probing the membrane with GAPDH (lower panel). It should be noted that some reduction in the level of total p65 resulted from the treatments with higher concentrations of NC1153 that could be due to decreased cellular viability at this time point. However, the lowest dose of NC1153 (10 μM) did not affect total p65 but reduced its Ser536 phosphorylation as well as BCL10 levels confirming that these reductions were not due to non-specific treatment effects.

### STAT5 depletion reduces BCL10 mRNA and protein expression, decreases the viability of Kit225 leukemia cells and diminishes NFκB DNA binding independently of IL-2 stimulation

**Figure 8:**
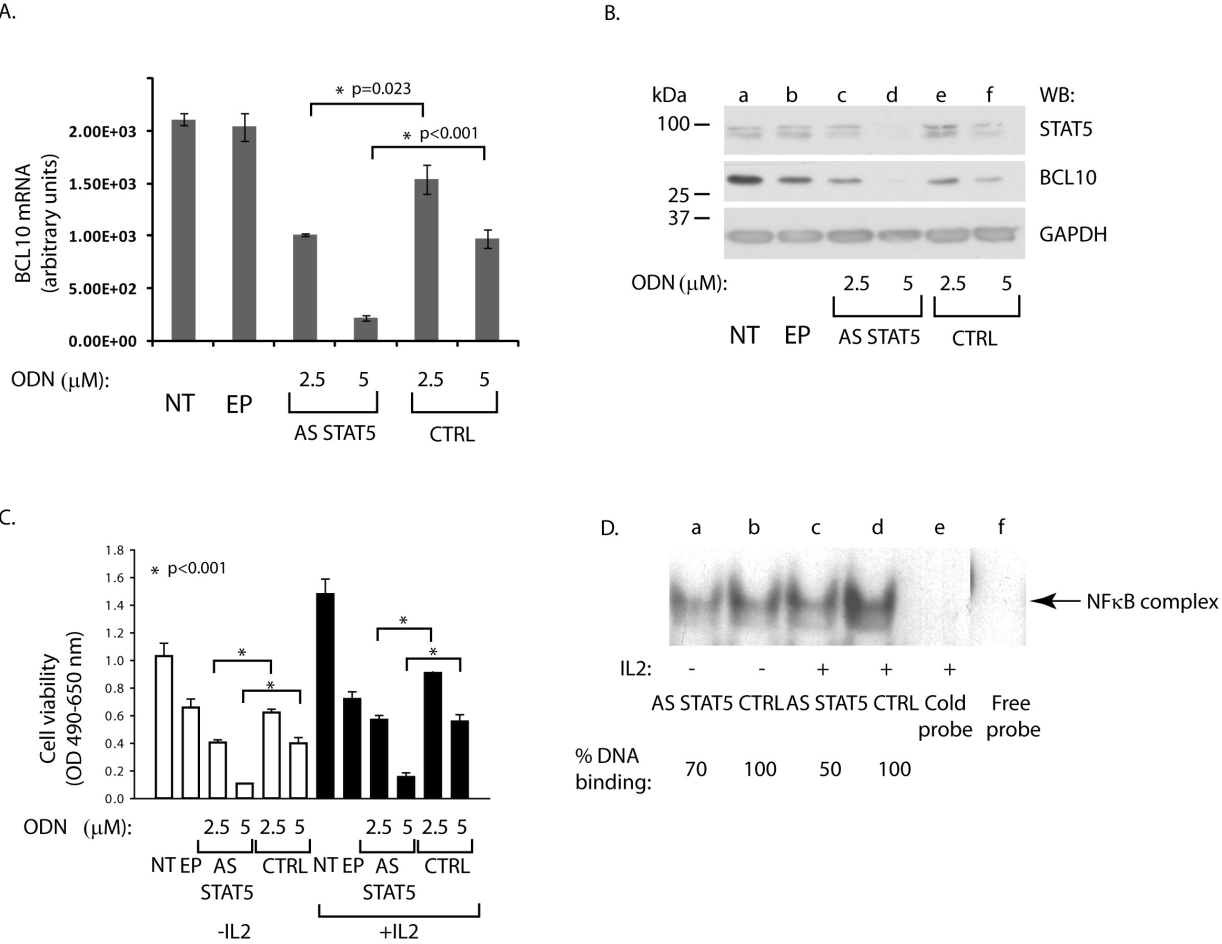
**(A) STAT5 depletion decreases BCL10 mRNA expression in a dose dependent manner**. Kit225 cells were treated with antisense STAT5 (AS STAT5) or control (CTRL) ODN as indicated and cultured for 24 h. Cells were harvested in duplicates. One pellet was used for total RNA isolation and cDNA preparation as described in the Methods. QRT-PCR was performed with primers specific to human BCL10. (B) **STAT5 depletion decreases BCL10 protein expression**. Parallel cell pellets were lysed and equal amounts (10 μg) of lysates resolved on 10% SDS-PAGE, then Western blotted with antibodies to BCL10, STAT5, followed by re-probing with antibodies to GAPDH (as a loading control). Representative data from two independent experiments are shown. (C) **STAT5 depletion decreases Kit225 cell viability**. Cell viability following electroporation (24 h) was assessed using MTS assay as described in the Methods. Cells were cultured either in the absence (white bars) or presence (black bars) of IL-2. Representative data from three independent experiments are shown. (* p < 0.001) (D) **Antisense STAT5 treatment decreases NFκB DNA binding**. Kit225 cells were electroporated with either AS STAT5 (AS STAT5) or control ODN (CTRL) and cultured in medium without (-) or with (+) IL-2 for 24 h. Nuclear proteins were isolated and incubated with [^32^P]-labeled NFκB probe (indicated to the right). Corresponding un-labeled NFκB probe in 100-fold molar excess (lane e) or free probe (lane f) was used to confirm the binding specificity. % DNA-binding was calculated by normalizing band intensities of STAT5 ODN treated samples to the corresponding CTRL ODN treated samples (100%). Representative data from two independent experiments are shown.

In order to test whether STAT5 has a direct role in regulating BCL10 expression and that this effect is independent of cytokines, antisense STAT5 ODN targeting both STAT5A and B were utilized. Earlier results demonstrated that STAT5 is a critical survival factor for activated T-cells and some lymphoid cell lines. [32] First, Kit225 cells were left untreated (Figure 8A and 8B, NT), electroporated without ODN (EP), with 2.5 or 5 μM antisense STAT5 ODN (AS STAT5) or 2.5 or 5 μM control ODN (CTRL), cultured in medium without (not shown) or with IL-2 for 24 h, and then harvested. Messenger RNA levels of BCL10 was measured via qRT PCR with primers specific to human *BCL10 *(Figure 8A) as described in the Methods. STAT5 depleted, but not control treated samples, displayed reduced BCL10 transcript levels. Next, parallel samples were lysed, equal amounts of lysates resolved on 12% SDS-PAGE and Western blotted with antibodies to BCL10 (Figure 8B). The blot was then re-probed with antibodies to STAT5 and GAPDH (as a loading control). Decreased STAT5 expression (Figure 8B, upper panel, lanes c-d) correlated with reduced BCL10 protein levels (middle panel) in a dose dependent manner, while GAPDH levels were not affected (lower panel). Kit225 cells depleted of STAT5 and cultured in the absence of IL-2 also displayed reduced BCL10 protein levels compared to controls (data not shown). Taken together, these data further support the notion that STAT5 regulates BCL10 expression.

Since STAT5 promotes lymphoid cell survival [32,33], cell viability following STAT5 depletion was also assessed by MTS assays. As shown in Figure 8C, reduced STAT5 and BCL10 expression decreased Kit225 cell viability in a dose dependent manner, regardless of the absence (white bars) or presence (black bars) of IL-2 in the culture medium. These data further suggest that non-cytokine activated STAT5 dependent gene regulation may be functionally important in tumor cell lines such as Kit225. Indeed, IL-2 starved Kit225 cells were greater than 90% viable (assessed by trypan blue exclusion) after 72 h, although tyrosine phosphorylated STAT5 was abolished within 24 h (data not shown). Interestingly, antisense oligonucleotide depletion of STAT5 resulted in greater than 50% reduction in cell viability within 24 h regardless of IL-2 (Figure 8C).

These results support the hypothesis that the cell survival promoting activities of STAT5 are, at least partially, cytokine independent and targets such as BCL10 may be responsible for this phenotype. To support this notice, the effect of STAT5 depletion on NFκB function was assessed. (It should be noted that a lower dose of antisense STAT5 ODN (2.5 μM) was employed within the present studies in order to avoid massive cellular death that follows STAT5 depletion.) Nuclear proteins were isolated from STAT5 antisense or CTRL (2.5 μM) ODN treated Kit225 cells at 24 h as described in the Methods and incubated with [^32^P]-labeled NFκB probe. The results presented in Figure 8D showed reduced constitutive DNA binding of NFκB following STAT5 depletion (lanes a, c) as compared to control ODN treated samples (lanes b, d). These data suggest that STAT5 regulates constitutive NFκB signaling in an IL-2-independent manner in Kit225 cells.

In summary, our results demonstrate that STAT5 mediated BCL10 expression occurs in the absence or presence of cytokine stimulation and STAT5 tyrosine phosphorylation. Moreover, these data indicate that STAT5 and NFκB pathways are interconnected and critical for regulating lymphoid/leukemic cancer cell proliferation/survival genes. The functional relevance of these findings is that therapeutic strategies that seek to disrupt cancer disease progression by blocking STAT tyrosine phosphorylation status alone may not prove effective and may be tumor or cell type dependent. Indeed, targeted disruption of tyrosine and non-tyrosine phosphorylated forms of STAT5 may both be required.

## Methods

### Cell culture and treatment

The human lymphoma cell lines YT [34] and Hut102 [35], the human T-cell line MT-2 [36], and leukemia cell line Kit225 [37] were maintained in RPMI-1640 medium containing 10% fetal calf serum, 2 mM L-glutamine and penicillin-streptomycin (50 IU/ml and 50 μg/ml, respectively). Kit225 media was supplemented with 20 U/ml human recombinant IL-2 (NCI Preclinical Repository). Prior to IL-2 stimulation, Kit225 cells were made quiescent for 24 h in their regular medium without IL-2. Cell stimulations were carried out with 10 nM IL-2. Antisense oligodeoxynucleotides (ODN) were synthesized by ISIS Pharmaceuticals, Inc. and used as previously described [38].

### Chromatin Immuno-precipitation

Chromatin immuno-precipitation was performed as previously described. [18] Chromatin was immuno-precipitated with either anti-STAT5A/B antibody (N-terminal, N-20, Santa Cruz Biotechnology Inc.); extreme C-terminal STAT5A and STAT5B mixture [39] or normal rabbit serum (IgG control) (Santa Cruz Biotechnology Inc.,) for 3 h at 4°C. DNA was recovered using Qiagen PCR Purification Kit and ultimately eluted with 100 μl 10 mM Tris pH 8.0. To confirm successful chromatin immuno-precipitation in Kit225 cells, PCR amplification of a known STAT5 binding element localized 5' to the human *IL2RA *gene within the Positive Regulatory Region III [19] (Forward: 5'-ACG TCT AGA AAG AAA GTG GTC-3' Reverse: 5'-CTG TCC CTG GAT GAA CCT AGT-3') was performed by quantitative real time PCR (40 cycles of 30 s at 94°C, 30 s at 50°C 30 s at 72°C) and 2× SYBR Green Master Mix from BioRad on a BioRad iQ5 qPCR machine. BCL10-SBR was amplified via qPCR with Forward: 5'-CCT GCC ATT ACC TTT GTC ATT AT-3' and Reverse: 5'-GGG AGT GTT CGA AAA ATG-3' primers. Values of transcripts in unknown samples were obtained by interpolating C_t _(PCR cycles to threshold) values on a standard curve. Standard curves were prepared from known amounts of purified, PCR-amplified DNA.

### Cloning of STAT5 DNA binding regions

The chromatin immuno-precipitated DNA was blunt ended by T4 DNA Polymerase (NEB, according to the manufacturer's recommendations) for 5 min at 37°C and recovered by purification with Qiagen's PCR Purification Kit. A unidirectional linker was annealed and ligated to the blunted DNA pool with T4 DNA ligase (Promega) as described earlier [40]. DNA was amplified using the linker as a primer to generate a sufficient amount to clone into the pCR II-TOPO vector using TOPO TA Cloning Kit with One Shot Max Efficiency DH5α-T1 E. coli according to the manufacturer's suggested protocol (Invitrogen). Competent E. coli cells were transformed by heat shock and plated on agarose plates containing ampicillin and S-gal (Sigma). White colonies were checked for DNA inserts by PCR with vector specific M13 primers performed directly on the colonies according to the manufacturer's protocol (Invitrogen) and visualized on ethidium-bromide stained 1% agarose gels. Positive colonies were amplified and plasmids purified with Qiagen's Miniprep Kit. The target DNA inserts were identified by DNA sequencing using vector specific M13 primers.

### Separation of cytosolic and nuclear proteins

Nuclear and cytoplasmic proteins were isolated by a protocol adapted from Panomics, Inc. for their Nuclear Extrcation Kit. Nuclear protein concentration was determined by BCA assay (Pierce), aliquoted and either used immediately to prepare samples for SDS-PAGE or stored at -70°C. Oligonucleotides corresponding to the *β-casein *gene promoter for STAT5 (5'-AGA TTT CTA GGA ATT CAA TCC-3') and NFκB consensus binding site (5'-AGT TGA GGG GAC TTT CCC AGG C-3') were obtained from Santa Cruz Biotechnology, Inc. and labeled with T4 Polynucleotide Kinase and [γ-^32^P]-ATP followed by ethanol precipitation. The nuclear extract/DNA binding mixtures were resolved on 5% native PAGE, dried and exposed to X-ray film.

### Electromobility Shift Assay and cold competition assay

EMSA was performed as described previously [41,42]. To validate the results of ChIP-cloning, randomly selected clones were amplified by PCR using vector specific M13 primers and the products isolated by the Qiagen PCR Purification Kit. DNA integrity was assessed using 1% agarose gel. The amplified inserts were used as cold competitors at 30–50-fold molar access in EMSA reactions using 5 μg IL-2-stimulated YT nuclear extracts and a [^32^P]-radiolabeled STAT5 DNA binding probe. As a positive control, cold competition was also performed with an amplified known STAT5 binding site located 5' to the human *CISH *gene (Forward: 5'-CTA TTG GCC CTC CCC GAC CG-3' Reverse: 5'-GGC GAG CTG CTG CCT AAT CC-3') [18] or *IL2RA *gene (primer sequences indicated above). The results were quantitated by Scion Image (Scion Corporation) or Un-Scan-It gel Version 6.1 (Silk Scientific Corporation) densitometry analysis software. Supershift analysis was performed with polyclonal anti-p65 and anti-p50 NFκB antibodies from Santa Cruz Biotechnology, Inc. by incubating the nuclear extract for 1 h at 4°C prior to the binding reactions.

### In silico Analyses

To determine the localization of the ChIP-cloned fragments, plasmids from the positive colonies were isolated and the inserts sequenced and located within the human genome by using the UCSC web-tool BLAT at  and Sanger Institute's Ensemble genome browser at . Proximal gene mapping of the genomic sequences up to 300 kb was performed using the Cis-Regulatory Element Annotation System (CEAS) at .

### Viability (MTS) assay

Cell viability was assessed with MTS reagent (Promega) in triplicates according to the manufacturer's instructions. Three independent experiments were performed. The error bars represent the standard deviation.

### Cell lysis and Western blotting

Cell lysis and Western blots with antibodies to JAK3, STAT5A or STAT5B were performed as previously described [42]. Monoclonal anti-phosphotyrosine STAT5 and anti-BCL10 antibodies were obtained from Millipore, monoclonal anti-STAT5 antibody from BD Biosciences, monoclonal anti-GAPDH antibody from RDI, monoclonal anti-Lamin A/C and polyclonal anti-p65 and anti-p50 NFκB antibodies from Santa Cruz Biotechnology, Inc., polyclonal anti-Ser536-p65 antibody from Cell Signaling, Inc. and all antibodies used at a dilution recommended by the manufacturer. Western blots were detected by enhanced chemiluminescence (ECL). For all samples, total protein was determined by the BCA method (Pierce).

### RNA isolation, cDNA synthesis and qRT-PCR

Total RNA was isolated from approximately 4–5 × 10^6 ^cells using the RNeasy kit (Qiagen), then DNase treated and quantitated by measuring OD at 260 nm; cDNA was synthesized with BioRad's iScript cDNA Synthesis Kit as recommended by the manufacturer (0.5 μg total RNA/each sample). Quantification based on real-time monitoring of amplification was determined using a BioRad iQ5 machine and 2× SYBR Green Mastermix (Biorad) with primers for human *BCL10 *(NM_003921) as follows: Forward: 5'-CCCGCTCCGCCTCCTCTCCTT-3', Reverse: 5'-GGCGCTTCTTCCGGGTCCG-3'. Relative numbers of mRNA molecules were normalized to 18S rRNA to correct for RNA concentration differences. Samples (cDNA corresponding to 5 ng total RNA/well) were run in triplicates in 25 μl reaction volumes with one control reaction containing no RT enzyme to test for potential DNA contamination. Values of transcripts in unknown samples were obtained by interpolating Ct (PCR cycles to threshold) values on a standard curve. Standard curves were prepared from serial dilution of non-treated Kit225 cDNA, with 10-fold differences, starting with cDNA corresponding to 62.5 ng total RNA/well to 6.25 pg total RNA/well. To ensure that fluorescent signals were specifically generated, a melting curve was obtained as recommended by BioRad.

### Plasmids and mutants

Expression plasmids for wild type and Y694F mutant mouse STAT5A were kindly provided by Dr. Hallgeir Rui and described in [27]. FLAG tagged versions of the cDNAs were subsequently created using pCMV-Tag2B vector (Stratagene), Hind III and Xho I cloning enzymes (NEB), Pfu Ultra High Fidelity DNA Polymerase (Stratagene) and T4 DNA Ligase (NEB). DNA amplification and purification steps were performed with Qiagen's Plasmid isolation and Purification Kits. All steps were carried out according to the manufacturers' recommendations. YT cells (2.5 million) were electroporated with an AMAXA Nucleofector^® ^and Cell Line Nucleofector^® ^Kit T, using 2 μg plasmid (1 μg pCMV-Tag2B and 1 μg pmaxGFP^®^) and program O-017, selected with 0.3 mg/ml G418 (Invitrogen) and sorted with a Beckman Coulter Epics Altra Cell Sorter.

### Statistical analyses

Normalized t-tests were performed using SigmaStat 3.1.

## Abbreviations

AS: antisense; BCL10: B-cell leukemia/lymphoma 10; CEAS: Cis-Regulatory Elememnt Annotation System; ChIP: chromatin immuno-precipitation; CISH: cytokine inducible SH2-containing protein [Homo sapiens]; ECL: enhanced chemiluminescence; EMSA: Electromobility Shift Assay; GAPDH: Glyceraldehyde 3-Phosphate Dehydrogenase; NFκB: Nuclear Factor kappa B; IL-2R: IL-2 Receptor; JAK: Janus Kinase; ODN: Oligodeoxynucleotide; PRR III: Positive Regulatory Region III; PY: phosphotyrosine; qRT-PCR: quantitative reverse transcriptase polymerase chain reaction; SIE: sis-inducible element; STAT: Signal Transducer and Activator of Transcription.

## Competing interests

The authors declare that they have no competing interests.

## Authors' contributions

ZSN designed, carried out experiments, interpreted and analyzed the results and wrote the manuscript, MJL participated in the ChIP experiments, JR assisted with the draft of the manuscript, AM generated the Y699F mutant of mSTAT5B-pCMVTag2B, HR participated in the design of the project and RAK designed the project and critically revised the manuscript. All authors read and approved the final manuscript.
